# Huperzine A: Is it an Effective Disease-Modifying Drug for Alzheimer’s Disease?

**DOI:** 10.3389/fnagi.2014.00216

**Published:** 2014-08-19

**Authors:** Zhong Ming Qian, Ya Ke

**Affiliations:** ^1^Laboratory of Neuropharmacology, Fudan University School of Pharmacy, Shanghai, China; ^2^School of Biomedical Sciences, Faculty of Medicine, The Chinese University of Hong Kong, Shatin, NT, Hong Kong, China

**Keywords:** huperzine A, Alzheimer’s disease, acetylcholinesterase inhibitor, disease-modifying agent, non-cholinergic effects

## Abstract

Alzheimer’s disease (AD) is a progressive neurodegenerative disorder for which there is no cure. Huperzine A (HupA) is a natural inhibitor of acetylcholinesterase (AChE) derived from the Chinese folk medicine *Huperzia serrata* (Qian Ceng Ta). It is a licensed anti-AD drug in China and is available as a nutraceutical in the US. A growing body of evidence has demonstrated that HupA has multifaceted pharmacological effects. In addition to the symptomatic, cognitive-enhancing effect via inhibition of AChE, a number of recent studies have reported that this drug has “non-cholinergic” effects on AD. Most important among these is the protective effect of HupA on neurons against amyloid beta-induced oxidative injury and mitochondrial dysfunction as well as via the up-regulation of nerve growth factor and antagonizing *N*-methyl-d-aspartate receptors. The most recent discovery that HupA may reduce brain iron accumulation lends further support to the argument that HupA could serve as a potential disease-modifying agent for AD and also other neurodegenerative disorders by significantly slowing down the course of neuronal death.

## Introduction

Huperzine A (HupA) is a natural inhibitor of acetylcholinesterase (AChE) derived from the Chinese folk medicine *Huperzia serrata* (Qian Ceng Ta) (Ratia et al., 2013). There is a long history of using *H. serrata* as a medicine in China to treat different kinds of disorders, including bruises, strains, swelling, rheumatism, schizophrenia, myasthenia gravis, and fever (Skolnick, 1997; Ma et al., 2007). HupA is a licensed anti-Alzheimer’s disease drug in China and is available as a nutraceutical in the US (Orhan et al., 2011). A growing body of evidence has demonstrated that HupA could effectively reverse or attenuate cognitive deficits in rodents, primates, and human (Howes and Perry, 2011). A recent systematic review and meta-analysis of randomized clinical trials concluded that HupA could improve cognitive function, daily living activity, and global clinical assessment in patients with Alzheimer’s disease (AD), with relatively few and mild adverse effects mainly related to its effect on the cholinergic system (Xing, 2014; Yang et al., 2014).

Currently, the pharmacological mechanisms of HupA in the treatment of AD have not been fully detailed. However, a number of recent studies have reported that this drug has “non-cholinergic” effects on AD (Ratia et al., 2013; Huang et al., 2014) in addition to the symptomatic, cognitive-enhancing effect of cholinesterase inhibition (Wang et al., 1986; Liu and Liu, 1995; Zhu and Giacobini, 1995). These new findings have greatly improved our understanding of pharmacological mechanisms of HupA in the treatment of AD. This article focuses on the recent advances in the studies on “non-cholinergic” roles of HupA. The updated understanding of the “cholinergic” action of HupA has recently been well-reviewed elsewhere (Fu and Li, 2011; Howes and Perry, 2011).

## Senile Plaques and Neurofibrillary Tangles: Consequences Rather than Initial Events

Alzheimer’s disease is a progressive neurological disorder clinically characterized by memory loss, mental deterioration, and impairment in the activities of daily living and behavioral disturbances throughout the disease course. The senile plaques and neurofibrillary tangles (NFTs), which are composed of self-polymerized amyloid-β peptide (Aβ) and hyperphosphorylated tau proteins, respectively, are the two major pathological hallmarks in AD brains (Zhao et al., 2009; Maeda et al., 2011). Although much progress has been made, the etiology of AD is still unclear and hence no preventive measure and effective disease-modifying treatment for this disease are currently available (Citron, 2010).

Accumulated data showed that senile plaques and NTFs are associated with the death of cholinergic neurons in AD. However, these two major pathological hallmarks might be just a consequence of the disease process rather than an initial event that causes AD (Huang et al., 2014). The failed result of several major clinical trials targeting Aβ (Ayton et al., 2012) is one of the strongest supports for this viewpoint. Doubtless, further studies on pathophysiological mechanisms involved in the formation of these two major pathological hallmarks in AD are fundamental and critical not only for elucidating the etiology of AD but also for the development of a disease-modifying treatment for AD. Also, further studies on the “non-cholinergic” of HupA might provide important insights into the etiology of AD.

## Non-Cholinergic Effects on Alzheimer’s Disease

A number of recent studies have reported that HupA has neuroprotective properties, possessing both “cholinergic” and “non-cholinergic” effects on AD. Here, we will discuss the major non-cholinergic effects of HupA on AD.

### Protecting neurons against Aβ-induced oxidative injury and apoptosis

Increased oxidative stress is associated with a number of neurodegenerative diseases, including AD. It has been well documented that Aβ treatment can generate oxidative stress that eventually triggers a state of neurotoxicity and cell death (Xiao et al., 2002). Studies have demonstrated that HupA could enhance the cell viability and the activities of antioxidant enzymes including glutathione peroxidase (GSH-Px), superoxide dismutase (SOD), and catalase (CAT); decrease the level of malondialdehyde (MDA) in PC12 (neuron-like rat pheochromocytoma) cells and cultured rat primary cortical neurons (Xiao et al., 2000a,b); and markedly reduced the MDA level in chronic cerebral hypo-perfusion rats (Wang et al., 2000) and aged rats (Shang et al., 1999). The protective effect of HupA on Aβ-induced cell lesion was also observed in NG108–15 cells (Zhang et al., 2002a). These results indicate that HupA can function as an antioxidant in Aβ-induced oxidative stress model by increasing the activities of antioxidant enzymes.

The neuroprotective effects of HupA against Aβ-induced oxidative injury are at least partly associated with its anti-apoptotic action. There is considerable evidence showing that Aβ can activate intracellular apoptosis pathways, which lead to neuronal death (Saille et al., 1999). Activation of caspase-3 is a key event in the execution of apoptotic cascade in central nervous system diseases. It has been demonstrated that HupA could attenuate the increase of caspase-3 activity induced by Aβ in cultured primary cortical neurons (Xiao et al., 2002) and inhibit Aβ-induced apoptosis by reversing the down-regulation of the expression of Bcl-2 and the up-regulation of the expressions of Bax and p53 (Wang et al., 2001a). HupA had the same effects on H_2_O_2_-induced apoptosis with a down-regulation of the bax and p53 genes and up-regulation of the Bcl-2 gene to normal levels (Wang et al., 2001b).

### Ameliorating mitochondrial malfunction in Alzheimer’s disease brain

Perturbations in mitochondrial function have long been observed in samples derived from clinically confirmed patients with AD, including altered mitochondrial morphology, compromised enzyme complexes in the tricarboxylic acid cycle, and reduced cytochrome *c* oxidase activity (Cardoso et al., 2001). In addition, accumulated evidence showed that mitochondria are direct targets of Aβ (Yao et al., 2011). Aβ could be accumulated within mitochondria and interact with a mitochondrial protein, Aβbinding-alcohol-dehydrogenase, resulting in decreased cytochrome *c* oxidase activity and increased oxidative stress (Reddy and Beal, 2008). The neurotoxicity induced by Aβ will trigger a vicious cycle in which excessive Aβ accumulation and sustained mitochondrial dysfunction synergize to activate a cascade of neurodegenerative pathways (Silva et al., 2012).

Studies have provided evidence that HupA has the ability to effectively ameliorate the mitochondrial malfunction. Aβ(25–35) treatment could lead to a rapid decline of adenosine 5’-triphosphate (ATP) level, an obvious disruption of mitochondrial membrane homeostasis and integrity, a reduction in key enzyme activities in the electron transport chain and the tricarboxylic acid cycle, and an increase in intracellular reactive oxygen species (ROS) in PC12 cells, while HupA not only attenuated these signs of cellular stress caused by Aβ, but also enhanced ATP concentration and decreased ROS accumulation (Gao and Tang, 2006). In isolated rat brain mitochondria, HupA was able to effectively prevent Aβ-induced mitochondrial swelling, ROS increase, and cytochrome *c* release. Furthermore, it could also ameliorate Aβ-induced decreases in mitochondrial respiration, ATP synthesis, mitochondrial respiratory chain enzyme activity, and transmembrane potential (Gao et al., 2009). There is no evidence for the existence of cholinergic system in isolated brain mitochondria. Therefore, the mitochondria-targeted effects of HupA are clearly independent of cholinergic system. In addition, HupA could inhibit the penetration of Aβ into mitochondria and ameliorated Aβ-induced dysfunction of tricarboxylic acid cycle in isolated brain cortical mitochondria (Yang et al., 2012).

### Antagonizing effects on *N*-methyl-d-aspartate receptors

Synaptic plasticity, the variable efficacy of neurotransmission at synapses, is thought to underlie the ability of the brain to store memories (Martin et al., 2000). The forms of synaptic plasticity that are most likely to be involved in memory storage require gene transcription and protein synthesis to stabilize synaptic changes over time (Adams and Dude, 2005). Glutamatergic synapses mediate virtually all excitatory neurotransmission in mammalian brains (Sucher et al., 1996). Glutamate released from presynaptic terminals activates several types of glutamate-gated ion channels on postsynaptic membranes, including *N*-methyl-d-aspartate (NMDA) receptors (Rao and Finkbeiner, 2007). Permeability to Ca^2+^ is a feature of all NMDA receptors (NMDA-R), which are composed of an essential NR1 subunit and multiple NR2 subunits (Rao and Finkbeiner, 2007). Excitotoxicity caused by disturbances of glutamatergic neurotransmission in the brain has been shown to be involved in the pathogenesis of AD (Emilien et al., 2000). NMDA receptor antagonists have been used as neuroprotective agents to ameliorate the cognition deficits of patients with AD (Emilien et al., 2000; Marx, 2000).

Huperzine A could inhibit NMDA-induced toxicity in a dose-dependent way in cultured primary neuronal cells (Marx, 2000). HupA interacts with the NMDA ion channel by inhibition of [^3^H]MK-801 and [^3^H]TCP binding in brain synaptosomal plasma membranes but not the glycine or NMDA ligand-specific sites. The non-competitive binding results suggest that HupA inhibits the NMDA-induced toxicity most likely by blocking NMDA ion channels and the subsequent Ca^2+^ mobilization at or near the PCP and MK-801 ligand sites (Gordon et al., 2001). HupA reversibly inhibited NMDA-induced current in acutely dissociated rat hippocampal pyramidal neurons and blocked specific [^3^H]MK-801 binding in synaptic membranes from rat cerebral cortex (Wang et al., 1999) and the inhibitory effect is non-competitive (Zhang and Hu, 2001). HupA acts as antagonist of the NMDA-R, acting at one of the polyamine binding sites on the NMDA-R (Zhang et al., 2002b).

### Regulating the expression and secretion of nerve growth factor

Nerve growth factor (NGF), a neurotrophin, plays a trophic role both during development and in adulthood (Aloe et al., 2012), and exerts its biological action by interacting with the specific receptor tropomyosin kinase receptor A (TrkA) (Huang and Reichard, 2003). Studies on rodents (Fischer et al., 1987) and primates (Tuszynski et al., 1991) have demonstrated that exogenous NGF was able to protect basal forebrain cholinergic neurons (BFCNs) from both traumatic insults and age-related cholinergic decline. Also, NGF is able to regulate both amyloid gene expression and protein processing (Rossner et al., 1998) and to counteract tau hyperphosphorylation (Zhang et al., 2010), acting directly at the two classical hallmarks of AD. A decreased NGF immunoreactivity in the BFCNs of patients with AD suggested that impaired NGF supply via retrograde transport could be the effective cause of cholinergic neurodegeneration in AD (Scott et al., 1995). These findings plus the experimental studies on NGF deficit-induced neurodegeneration in transgenic mice demonstrated a novel causal link between neurotrophic signaling deficits and Alzheimer’s neurodegeneration (Cattaneo and Calissano, 2012). Alteration in the homeostasis and equilibrium of NGF processing and NGF/TrkA signaling in target neurons has been considered as an upstream driver of all the cellular and molecular central hallmarks of AD (Tang et al., 2005).

Therefore, it will be very important to re-establish a correct homeostatic balance between ligands, and receptors, of the NGF pathway (Tang et al., 2005). Wang et al. (2006) investigated the effects of HupA on secretion of NGF in cultured rat cortical astrocytes and neurite outgrowth in rat PC12 cells and demonstrated that HupA treatment induced a significant increase in both mRNA and protein levels of NGF in astrocytes. They also found that treatment of PC12 cells with HupA led to a significant increase in the number of neurite-bearing cells without significant alteration in cell viability or other signs of cytotoxicity. The positive effect of HupA on the mRNA and protein levels of NGF, accompanied by the inhibitory effects on the memory deficits and neuronal damage, have also been reported in mice treated with transient cerebral ischemia and re-perfusion (Wang et al., 2006). These findings suggested that HupA has a direct or indirect neurotrophic activity, which might be beneficial in treatment of neurodegenerative disorders such as AD (Tang et al., 2005).

### Promoting non-amyloidogenic amyloid precursor protein processing by activating protein kinase C and the Wnt/β-catenin signaling pathway

The canonical Wnt signaling pathway has an important role in development and maintenance of the nervous system and is also associated with neurodegenerative diseases (De Ferrari et al., 2003). Loss of Wnt signaling function is involved in Aβ-dependent neurodegeneration in the AD brain and two key components of the canonical Wnt signaling pathway, glycogen synthase kinase (GSK)-3β and β-catenin, are altered in the AD model mouse brain (Pei et al., 1999). It has also been reported that activation of Wnt signaling can prevent neurodegeneration induced by Aβ fibrils (De Ferrari et al., 2003) and that inhibition of GSK-3β and enhancement of β-catenin activity could prevent the loss of function of the Wnt signaling pathway caused by Aβ toxicity (Farias et al., 2005).

Wang et al. (2011) showed that HupA has a role in the regulation of Wnt signaling in APPswe/PS1dE9 (APP/PS1) transgenic mouse and APPsw cell models and could significantly inhibit GSK-3β activity and stabilize β-catenin protein level *in vivo* and *in vitro*. They also found that HupA induced a significant increase in the phosphorylation levels of both GSK-3α and GSK-3β proteins in APP/PS1 mouse brain and APPsw-overexpressing cells, while activation of both GSK-3α and GSK-3β through their autophosphorylation has been implicated in AD pathogenesis (Caricasole et al., 2004). Their findings suggested that HupA can inhibit the activity of GSK-3α/β and, hence, may inhibit Aβ generation and tau phosphorylation. HupA reverses protein kinase C (PKC)- and Wnt-inhibitor-induced inhibition of non-amyloidogenic processing of amyloid precursor protein (APP), paralleled by an inactivation of GSK-3 and a reversal of the level of β-catenin (Zhang et al., 2004; Wang et al., 2011). These findings imply that HupA is involved in regulation of the Wnt signaling pathway and that HupA regulates APP processing to the non-amyloidogenic pathway at least partly through activation of PKC and Wnt/β-catenin signaling.

### Reducing iron in AD brain

Misregulation in brain iron has been considered to be one of the primary causes of neuronal death in neurodegenerative disorders (Qian et al., 1997; Qian and Shen, 2001; Lei et al., 2012). Evidence has also been gathered to imply that Aβ production, precipitation, and toxicity in AD are caused by abnormal interactions with neocortical iron (Zhu et al., 2004; Zhao et al., 2008; Smith et al., 2010). In a recent study (Huang et al., 2014), we demonstrated for the first time that HupA was able to reduce significantly the contents of insoluble and soluble A(β-40 and A(β-42 and hyperphosphorylated tau in the brain of APP/PS1 transgenic mice. Also, HupA could decrease the deposition of amyloid plaques and the levels of oligomeric Aβ. It also suppressed APP695 expression and increase ADAM10 contents in APP/PS1 mice brain. However, all of these beneficial effects of HupA could be largely abolished by high iron diet. In addition, the iron levels in the hippocampus and cortex of the APP/PS1 mice were found to be elevated while quantitative analysis revealed that the abnormal increase in brain iron contents in these two regions were almost completely normalized by the chronic treatment with HupA. Our findings provided direct evidence for the inhibitory effect of HupA on brain iron, and implied that the beneficial effects of HupA on AD is caused by the reduction in brain iron in the APP/PS1 mice.

Furthermore, we showed that mutation of APPswe/PS1dE9 could lead to a significant increase in transferrin receptor 1 (TfR1) expression and that HupA was able to induce a significant reduction in TfR1 expression in the brain of APP/PS1 mice *in vivo*, and a remarkable reduction in TfR1 expression as well as transferrin-bound iron (TBI) uptake in the cultured neurons *in vitro*. Two AChE inhibitors donepezil or galantamine did not induce any changes in TfR1 expression in the brain and had no effect on TBI uptake by the neurons, indicating that effects of HupA on TfR1 expression and TBI uptake were independent of its anti-AChE action. Based on the above findings, we propose that HupA has the ability to inhibit TfR1 expression and then reduce TBI uptake by the neurons or other brain cells, leading to a progressive reduction in iron contents and also iron-induced oxidative stress in the brain (Huang et al., 2014).

## Disease-Modifying Potential of Huperzine A

Currently Hup A is proved to be used in China to improve symptoms of AD (Zangara, 2003). The information available from clinical trials of Hup A was mainly on patients with mild and moderate AD, showing significant improvement in their cognitive functions (Rafii et al., 2011; Yang et al., 2014). However, due to the relatively short study duration (<6 months) and lack of other pathological assessments, it is still premature to conclude from these trials that HupA could slow down the progress of AD. On the other hand, studies on animal model of AD (e.g., APP/PS1 mice) have shown that HupA could suppress Aβ accumulation, and amyloid plaques and hyperphosphorylated tau formation when drug administration began at an early stage (Wang et al., 2011; Huang et al., 2014). This result implied that HupA may be also beneficial if taken at preclinical stage of AD. Therefore, further long-term longitudinal clinical trials and preclinical studies are demanded in determining the optimal time to start the treatment and monitoring of disease progression. Additional studies will also be needed to address the safety of long-term treatment.

## Summary

**Figure 1:**
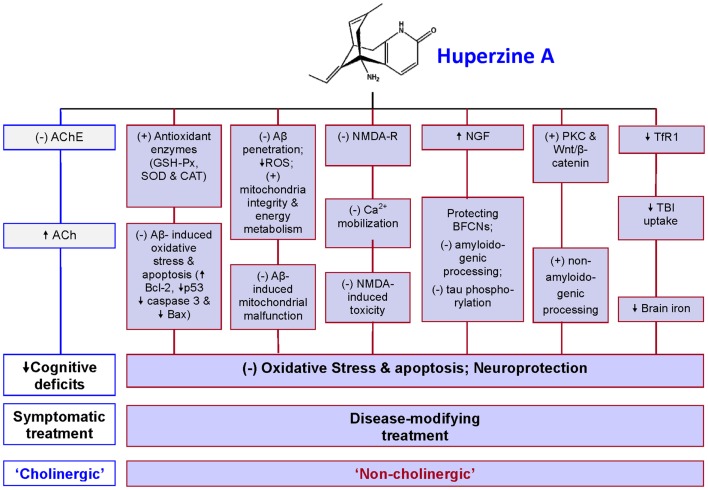
**A summary of pharmacological mechanisms of huperzine A (HupA) in the treatment of Alzheimer’s disease (AD)**. In addition to acting as an acetylcholinesterase (AchE) inhibitor, HupA has non-cholinergic roles in the treatment of AD to protect neurons and other brain cells from oxidative stress damage and apoptosis. It has been demonstrated that HupA has the ability: (1) to protect neurons against Aβ-induced oxidative injury and apoptosis by enhancing the activities of antioxidant enzymes including glutathione peroxidase (GSH-Px), superoxide dismutase (SOD), and catalase (CAT), attenuating the Aβ-induced increase in caspase-3 activity, and inhibiting Aβ-induced apoptosis by reversing the down-regulation of the expression of Bcl-2 and the up-regulation of Bax and p53 expressions; (2) to ameliorate mitochondrial malfunction in AD brain by preventing Aβ-penetration into the mitochondria, suppressing ROS production, and improving mitochondrial integrity and energy metabolism, thus minimizing Aβ-induced mitochondrial malfunction; (3) to act as antagonist of the NMDA receptors (NMDA-R) to inhibit the NMDA-induced toxicity by blocking NMDA ion channels and the subsequent Ca^2+^ mobilization; (4) to increase nerve growth factor (NGF), which protects basal forebrain cholinergic neurons (BFCNs) from both traumatic insults and age-related cholinergic decline and regulate both amyloid gene expression and protein processing and counteract tau hyperphosphorylation, acting directly at the two classical hallmarks of AD; (5) to promote non-amyloidogenic processing by activating protein kinase C (PKC) and the Wnt/β-catenin signaling pathway; and (6) to inhibit transferrin receptor 1 (TfR1) expression and then reduce transferrin-bound iron (TBI) uptake by the neurons or other brain cells, which have TfR1 expression on the membrane, leading to a progressive reduction in iron contents and also iron-induced oxidative stress in the brain. Based on these roles, it is reasonable to consider HupA as an effective disease-modifying drug for AD. (+) = stimulate; (−) = inhibit; ↑ = increase; ↓ = decrease; Ach = acetylcholine.

Studies on non-cholinergic roles of HupA have made important contributions for the understanding of pharmacological mechanisms of HupA in the treatment of AD. In addition to the symptomatic, cognitive-enhancing effect via cholinesterase inhibition, HupA has a number of “non-cholinergic” effects on AD, including the ability to protect neurons against Aβ-induced oxidative injury and apoptosis, to ameliorate mitochondrial malfunction, to antagonize NMDA-R, to regulate NGF, promote non-amyloidogenic APP processing, and to reduce iron in the brain (Figure 1). Therefore, this multiplicity of action renders HupA a highly effective drug and potentially it could serve as a disease-modifying drug for AD. It may also be used for the treatment of other neurodegenerative disorders although further studies are needed.

## Author Contributions

Ya Ke and Zhong Ming Qian analyzed the data and wrote the paper.

## Conflict of Interest Statement

The authors declare that the research was conducted in the absence of any commercial or financial relationships that could be construed as a potential conflict of interest.

## Abbreviations

Aβ, amyloid-β peptide; AChE, acetylcholinesterase; AD, Alzheimer’s disease; APP, amyloid precursor protein; APP/PS1, APPswe/PS1dE9; ATP, adenosine 5’-triphosphate; BFCNs, basal forebrain cholinergic neurons; CAT, catalase; GSH-Px, glutathione peroxidase; GSK-3, glycogen synthase kinase-3; H_2_O_2_, hydrogen peroxide; HupA, huperzine A; MDA, malondialdehyde; NFTs, neurofibrillary tangles; NGF, nerve growth factor; NMDA, *N*-methyl-d-aspartate; PKC, protein kinase C; ROS, reactive oxygen species; SOD, superoxide dismutase; TBI, transferrin-bound iron; TfR1, transferrin receptor 1; TrkA, tropomyosin kinase receptor A.

## References

[B1] Adams J. P. Dude S. M. (2005). Late-phase long-term potentiation: getting to the nucleus. Nat. Rev. Neurosci. 6, 737–743 10.1038/nrn1749 16136174

[B2] Aloe L. Rocco M. L. Branching P. Mani L. (2012). Nerve growth factor: from the early discoveries to the potential clinical use. J. Transl. Med. 10, 239 10.1186/1479-5876-10-239 23190582 PMC3543237

[B3] Ayton S. Lei P. Bush A. I. (2012). Homeostasis in Alzheimer’s disease. Free Radic. Biol. Med. 62, 76–89 10.1016/j.freeradbiomed.2012.10.558 23142767

[B4] Cardoso S. M. Santos S. Swerdlow R. H. Oliveira C. R. (2001). Functional mitochondria are required for amyloid beta-mediated neurotoxicity. FASEB J. 15, 1439–1441 11387250 10.1096/fj.00-0561fje

[B5] Caricasole A. Copani A. Caraci F. Aronica E. Rozemuller A. J. Caruso A. (2004). Induction of Dickkopf-1, a negative modulator of the Wnt pathway, is associated with neuronal degeneration in Alzheimer’s brain. J. Neurosci. 24, 6021–6027 10.1523/JNEUROSCI.1381-04.2004 15229249 PMC6729239

[B6] Cattaneo A. Calissano P. (2012). Nerve growth factor and Alzheimer’s disease: new facts for an old hypothesis. Mol. Neurobiol. 46, 588–604 10.1007/s12035-012-8310-9 22940884

[B7] Citron M. (2010). Alzheimer’s disease: strategies for disease modification. Nat. Rev. Drug Discov. 9, 387–398 10.1038/nrd2896 20431570

[B8] De Ferrari G. V. Chacon M. A. Barria M. I. Garrido J. L. Godoy J. A. Olivares G. (2003). Activation of Wnt signaling rescues neurodegeneration and behavioral impairments induced by beta-amyloid fibrils. Mol. Psychiatry 8, 195–208 10.1038/sj.mp.4001208 12610652

[B9] Emilien G. Beyreuther K. Masters C. L. Maloteaux J. M. (2000). Prospects for pharmacological intervention in Alzheimer disease. Arch. Neurol. 57, 454–459 10.1001/archneur.57.4.454 10768617

[B10] Farias G. G. Godoy J. A. Vazquez M. C. Adani R. Meshulam H. Avila J. (2005). The anti-inflammatory and cholinesterase inhibitor bifunctional compound IBU-PO protects from beta-amyloid neurotoxicity by acting on Wnt signaling components. Neurobiol. Dis. 18, 176–183 10.1016/j.nbd.2004.09.012 15649708

[B11] Fischer W. Wictorin K. Bjorklund A. Williams L. R. Varon S. Gage F. H. (1987). Amelioration of cholinergic neuron atrophy and spatial memory impairment in aged rats by nerve growth factor. Nature 329, 65–68 10.1038/329065a0 3627243

[B12] Fu L. M. Li J. T. (2011). A systematic review of single Chinese herbs for Alzheimer’s disease treatment. Evid. Based Complement. Alternat. Med. 2011, 640284 10.1093/ecam/nep136 19737808 PMC3136754

[B13] Gao X. Tang X. C. (2006). Huperzine A attenuates mitochondrial dysfunction in beta-amyloid-treated PC12 cells by reducing oxygen free radicals accumulation and improving mitochondrial energy metabolism. J. Neurosci. Res. 83, 1048–1057 10.1002/jnr.20791 16493671

[B14] Gao X. Zheng C. Y. Yang L. Tang X. C. Zhang H. Y. (2009). Huperzine A protects isolated rat brain mitochondria against beta-amyloid peptide. Free Radic. Biol. Med. 46, 1454–1462 10.1016/j.freeradbiomed.2009.02.028 19272446

[B15] Gordon R. K. Nigam S. V. Weitz J. A. Dave J. R. Doctor B. P. Ved H. S. (2001). The NMDA receptor ion channel: a site for binding of huperzine A. J. Appl. Toxicol. 21(Suppl. 1), S47–S51 10.1002/jat.805 11920920

[B16] Howes M. J. Perry E. (2011). The role of phytochemicals in the treatment and prevention of dementia. Drugs Aging 28, 439–468 10.2165/11591310-000000000-00000 21639405

[B17] Huang E. J. Reichard L. F. (2003). TRK receptors: roles in neuronal signal transduction. Annu. Rev. Biochem. 72, 609–642 10.1146/annurev.biochem.72.121801.161629 12676795

[B18] Huang X. T. Qian Z. M. He X. Gong Q. Wu K. C. Jiang L. R. (2014). Reducing iron in the brain: a novel pharmacologic mechanism of huperzine A in the treatment of Alzheimer’s disease. Neurobiol. Aging 35, 1045–1054 10.1016/j.neurobiolaging.2013.11.004 24332448

[B19] Lei P. Ayton S. Finkelstein D. I. Spoerri L. Ciccotosto G. D. Wright D. K. (2012). Tau deficiency induces parkinsonism with dementia by impairing APP-mediated iron export. Nat. Med. 18, 291–295 10.1038/nm.2613 22286308

[B20] Liu M. Y. Liu H. C. (1995). Intelligence promoting Chinese materia medica. [Article in Chinese]. Zhongguo Zhong Xi Yi Jie He Za Zhi 15, 59–61 7767069

[B21] Ma X. Tan C. Zhu D. Gang D. R. Xiao P. (2007). Huperzine A from *Huperzia* species – an ethnopharmacolgical review. J. Ethnopharmacol. 113, 15–34 10.1016/j.jep.2007.05.030 17644292

[B22] Maeda J. Zhang M. R. Okauchi T. Ji B. Ono M. Hattori S. (2011). In vivo positron emission tomographic imaging of glial responses to amyloid-beta and tau pathologies in mouse models of Alzheimer’s disease and related disorders. J. Neurosci. 31, 4720–4730 10.1523/JNEUROSCI.3076-10.2011 21430171 PMC3251921

[B23] Martin S. J. Grimwood P. D. Morris R. G. (2000). Synaptic plasticity and memory: an evaluation of the hypothesis. Annu. Rev. Neurosci. 23, 649–711 10.1146/annurev.neuro.23.1.649 10845078

[B24] Marx J. (2000). Alzheimer’s congress. Drug shows promise for advanced disease. Science 289, 375–377 10.1126/science.289.5478.375b 10939942

[B25] Orhan I. E. Orhan G. Gurkas E. (2011). An overview on natural cholinesterase inhibitors – a multi-targeted drug class – and their mass production. Mini Rev. Med. Chem. 11, 836–842 10.2174/138955711796575434 21762104

[B26] Pei J. J. Braak E. Braak H. Grundke-Iqbal I. Iqbal K. Winblad B. (1999). Distribution of active glycogen synthase kinase 3beta (GSK-3beta) in brains staged for Alzheimer disease neurofibrillary changes. J. Neuropathol. Exp. Neurol. 58, 1010–1019 10.1097/00005072-199909000-00011 10499443

[B27] Qian Z. M. Shen X. (2001). Brain iron transport and neurodegeneration. Trends Mol. Med. 7, 103–110 10.1016/S1471-4914(00)01910-9 11286780

[B28] Qian Z. M. Wang Q. Pu Y. (1997). Brain iron and neurological disorders. Chin. Med. J. 110, 455–458 9594247

[B29] Rafii M. S. Walsh S. Little J. T. Behan K. Reynolds B. Ward C. (2011). A phase II trial of huperzine A in mild to moderate Alzheimer disease. Neurology 76, 1389–1394 10.1212/WNL.0b013e318216eb7b 21502597 PMC3269774

[B30] Rao V. R. Finkbeiner S. (2007). NMDA and AMPA receptors: old channels, new tricks. Trends Neurosci. 30, 284–291 10.1016/j.tins.2007.03.012 17418904

[B31] Ratia M. Giménez-Llort L. Camps P. Muñoz-Torrero D. Pérez B. Clos M. V. (2013). Huprine X and huperzine A improve cognition and regulate some neurochemical processes related with Alzheimer’s disease in triple transgenic mice (3xTg-AD). Neurodegener. Dis. 11, 129–140 10.1159/000336427 22626981

[B32] Reddy P. H. Beal M. F. (2008). Amyloid beta, mitochondrial dysfunction and synaptic damage: implications for cognitive decline in aging and Alzheimer’s disease. Trends Mol. Med. 14, 45–53 10.1016/j.molmed.2007.12.002 18218341 PMC3107703

[B33] Rossner S. Ueberham U. Schliebs R. Perez-Polo J. R. Bigl V. (1998). The regulation of amyloid precursor protein metabolism by cholinergic mechanisms and neurotrophin receptor signaling. Prog. Neurobiol. 56, 541–569 10.1016/S0301-0082(98)00044-6 9775403

[B34] Saille C. Marin P. Martinou J. C. Nicole A. London J. Ceballos-Picot I. (1999). Transgenic murine cortical neurons expressing human Bcl-2 exhibit increased resistance to amyloid beta-peptide neurotoxicity. Neuroscience 92, 1455–1463 10.1016/S0306-4522(99)00089-5 10426499

[B35] Scott S. A. Mufson E. J. Weingartner J. A. Skau K. A. Crutcher K. A. (1995). Nerve growth factor in Alzheimer’s disease: increased levels throughout the brain coupled with declines in nucleus basalis. J. Neurosci. 15, 6213–6221 7666203 10.1523/JNEUROSCI.15-09-06213.1995 PMC6577665

[B36] Shang Y. Z. Ye J. W. Tang X. C. (1999). Improving effects of huperzine A on abnormal lipid peroxidation and superoxide dismutase in aged rats. Zhongguo Yao Li Xue Bao 20, 824–828 11245091

[B37] Silva D. F. Selfridge J. E. Lu J. H. Lezi E. Cardoso S. M. Swerdlow R. H. (2012). Mitochondrial abnormalities in Alzheimer’s disease: possible targets for therapeutic intervention. Adv. Pharmacol. 64, 83–126 10.1016/B978-0-12-394816-8.00003-9 22840745 PMC3625400

[B38] Skolnick A. A. (1997). Old Chinese herbal medicine used for fever yields possible new Alzheimer disease therapy. JAMA 277, 776 10.1001/jama.1997.03540340010004 9052690

[B39] Smith M. A. Zhu X. Tabaton M. Liu G. McKeel D. W. Jr. Cohen M. L. (2010). Increased iron and free radical generation in preclinical Alzheimer disease and mild cognitive impairment. J. Alzheimers Dis. 19, 363–372 10.3233/JAD-2010-1239 20061651 PMC2842004

[B40] Sucher N. J. Awobuluyi M. Choi Y. B. Lipton S. A. (1996). NMDA receptors: from genes to channels. Trends Pharmacol. Sci. 17, 348–355 10.1016/S0165-6147(96)10046-8 8979769

[B41] Tang L. L. Wang R. Tang X. C. (2005). Effects of huperzine A on secretion of nerve growth factor in cultured rat cortical astrocytes and neurite outgrowth in rat PC12 cells. Acta Pharmacol. Sin. 26, 673–678 10.1111/j.1745-7254.2005.00130.x 15916732

[B42] Tuszynski M. H. Sang H. Yoshida K. Gage F. H. (1991). Recombinant human nerve growth factor infusions prevent cholinergic neuronal degeneration in the adult primate brain. Ann. Neurol. 30, 625–636 10.1002/ana.410300502 1763889

[B43] Wang C. Y. Zheng W. Wang T. Xie J. W. Wang S. L. Zhao B. L. (2011). Huperzine A activates Wnt/β-catenin signaling and enhances the nonamyloidogenic pathway in an Alzheimer transgenic mouse model. Neuropsychopharmacology 36, 1073–1089 10.1038/npp.2010.245 21289607 PMC3077275

[B44] Wang L. M. Han Y. F. Tang X. C. (2000). Huperzine A improves cognitive deficits caused by chronic cerebral hypoperfusion in rats. Eur. J. Pharmacol. 398, 65–72 10.1016/S0014-2999(00)00291-0 10856449

[B45] Wang R. Xiao X. Q. Tang X. C. (2001a). Huperzine A attenuates hydrogen peroxide-induced apoptosis by regulating expression of apoptosis-related genes in rat PC12 cells. Neuroreport 12, 2629–2634 10.1097/00001756-200108280-00009 11522938

[B46] Wang R. Zhang H. Y. Tang X. C. (2001b). Huperzine A attenuates cognitive dysfunction and neuronal degeneration caused by beta-amyloid protein-(1–40) in rat. Eur. J. Pharmacol. 421, 149–156 10.1016/S0014-2999(01)01030-5 11516430

[B47] Wang X. D. Zhang J. M. Yang H. H. Hu G. Y. (1999). Modulation of NMDA receptor by huperzine A in rat cerebral cortex. Acta Pharmacol. Sin. 20, 31–35 10437121

[B48] Wang Y. E. Yue D. X. Tang X. C. (1986). Anti-cholinesterase activity of huperzine A. [Article in Chinese]. Zhongguo Yao Li Xue Bao 7, 110–113 2946143

[B49] Wang Z. F. Tang L. L. Yan H. Wang Y. J. Tang X. C. (2006). Effects of huperzine A on memory deficits and neurotrophic factors production after in mice. Pharmacol. Biochem. Behav. 83, 603–611 10.1016/j.pbb.2006.03.027 16687166

[B50] Xiao X. Q. Wang R. Han Y. F. Tang X. C. (2000a). Protective effects of huperzine A on beta-amyloid(25–35) induced oxidative injury in rat pheochromocytoma cells. Neurosci. Lett. 286, 155–158 10.1016/S0304-3940(00)01088-0 10832008

[B51] Xiao X. Q. Wang R. Tang X. C. (2000b). Huperzine A and tacrine attenuate beta-amyloid peptide-induced oxidative injury. J. Neurosci. Res. 61, 564–569 10.1002/1097-4547(20000901)61:5<564::AID-JNR11>3.3.CO;2-O 10956426

[B52] Xiao X. Q. Zhang H. Y. Tang X. C. (2002). Huperzine A attenuates amyloid beta-peptide fragment 25–35-induced apoptosis in rat cortical neurons via inhibiting reactive oxygen species formation and caspase-3 activation. J. Neurosci. Res. 67, 30–36 10.1002/jnr.10075 11754078

[B53] Xing S. H. Zhu C. X. Zhang R. An L. (2014). Huperzine a in the treatment of Alzheimer’s disease and vascular dementia: a meta-analysis. Evid. Based Complement Alternat. Med. 2014, 363985 10.1155/2014/363985 24639880 PMC3930088

[B54] Yang G. Wang Y. Tian J. Liu J. P. (2014). Huperzine a for Alzheimer’s disease: a systematic review and meta-analysis of randomized clinical trials. PLoS ONE 8:e74916 10.1371/journal.pone.0074916 24086396 PMC3781107

[B55] Yang L. Ye C. Y. Huang X. T. Tang X. C. Zhang H. Y. (2012). Decreased accumulation of subcellular amyloid-beta with improved mitochondrial function mediates the neuroprotective effect of huperzine A. J. Alzheimers Dis. 31, 131–142 10.3233/JAD-2012-120274 22531425

[B56] Yao J. Rettberg J. R. Klosinski L. P. Cadenas E. Brinton R. D. (2011). Shift in brain metabolism in late onset Alzheimer’s disease: implications for biomarkers and therapeutic interventions. Mol. Aspects Med. 32, 247–257 10.1016/j.mam.2011.10.005 22024249 PMC3658304

[B57] Zangara A. (2003). The psychopharmacology of huperzine A: an alkaloid with cognitive enhancing and neuroprotective properties of interest in the treatment of Alzheimer’s disease. Pharmacol. Biochem. Behav. 75, 675–686 10.1016/S0091-3057(03)00111-4 12895686

[B58] Zhang H. Y. Liang Y. Q. Tang X. C. He X. C. Bai D. L. (2002a). Stereoselectivities of enantiomers of huperzine A in protection against amyloid 25–35-induced injury in PC12 and NG108–15 cells and cholinesterase inhibition in mice. Neurosci. Lett. 317, 143–146 10.1016/S0304-3940(01)02437-5 11755260

[B59] Zhang Y. H. Zhao X. Y. Chen X. Q. Wang Y. Yang H. H. Hu G. Y. (2002b). Spermidine antagonizes the inhibitory effect of huperzine A on [3H]dizocilpine (MK-801) binding in synaptic membrane of rat cerebral cortex. Neurosci. Lett. 319, 107–110 10.1016/S0304-3940(01)02565-4 11825682

[B60] Zhang H. Y. Yan H. Tang X. C. (2004). Huperzine A enhances the level of secretory amyloid precursor protein and protein kinase C-alpha in intracerebroventricular beta-amyloid-(1–40) infused rats and human embryonic kidney 293 Swedish mutant cells. Neurosci. Lett. 360, 21–24 10.1016/j.neulet.2004.01.055 15082169

[B61] Zhang J. M. Hu G. Y. (2001). Huperzine A, a nootropic alkaloid, inhibits *N*-methyl-d-aspartate-induced current in rat dissociated hippocampal neurons. Neuroscience 105, 663–669 10.1016/S0306-4522(01)00206-8 11516831

[B62] Zhang Z. H. Xi G. M. Li W. C. Ling H. Y. Qu P. Fang X. B. (2010). Cyclic-AMP response element binding protein and tau are involved in the neuroprotective mechanisms of nerve growth factor during focal cerebral ischemia/reperfusion in rats. J. Clin. Neurosci. 17, 353–356 10.1016/j.jocn.2009.07.086 20071183

[B63] Zhao L. Qian Z. M. Zhang C. Wing H. Y. Du F. Ya K. (2008). Amyloid beta-peptide 31-35-induced neuronal apoptosis is mediated by caspase-dependent pathways via cAMP-dependent protein kinase A activation. Aging Cell 7, 47–57 10.1111/j.1474-9726.2007.00352.x 18005252

[B64] Zhao L. Zhao S. T. Qian Z. M. Zhang C. Wu X. M. Du F. (2009). Activation of group III metabotropic glutamate receptor reduces intracellular calcium in beta-amyloid peptide [31-35]-treated cortical neurons. Neurotox. Res. 16, 174–183 10.1007/s12640-009-9068-3 19526293

[B65] Zhu X. Raina A. K. Perry G. Smith M. A. (2004). Alzheimer’s disease: the two-hit hypothesis. Lancet Neurol. 3, 219–226 10.1016/S1474-4422(04)00707-0 15039034

[B66] Zhu X. D. Giacobini E. (1995). Second generation cholinesterase inhibitors: effect of (L)-huperzine-A on cortical biogenic amines. J. Neurosci. Res. 41, 828–835 10.1002/jnr.490410613 7500384

